# In-Person Versus Online Training in Simulations of Helping Babies Breathe: A Randomized Controlled Trial

**DOI:** 10.7759/cureus.64677

**Published:** 2024-07-16

**Authors:** Peter Kfoury, Faouzi Maalouf, Fatima Nasser, Talin Gulgulian, Lama Charafeddine

**Affiliations:** 1 Faculty of Medicine, American University of Beirut Medical Center, Beirut, LBN; 2 Pediatrics and Adolescent Medicine, American University of Beirut Medical Center, Beirut, LBN; 3 Maternal and Child Health, Rafic Hariri School of Nursing, American University of Beirut Medical Center, Beirut, LBN

**Keywords:** tele-intensive care, virtual medicine, neonatal resuscitation, online debriefing, helping babies breathe

## Abstract

Background: Birth asphyxia is a leading cause of neonatal deaths, but simple interventions may prevent it. The Helping Babies Breathe (HBB) course has significantly reduced neonatal mortality rates in lower and middle-income countries (LMICs) by training healthcare providers (i.e. midwives and nurses) on the essential skills of bag-and-mask ventilation and postnatal care. Although several studies have supported the efficacy of virtual learning in other medical education programs, there is still a lack of knowledge regarding a virtual approach to HBB. This study aims to compare the effectiveness of online versus in-person learning of the HBB course among medical and nursing students.

Methods: The study is a two-arm parallel randomized non-inferiority controlled trial, that includes medical and nursing students. Participants were randomly assigned to either online or in-person debriefing during the hands-on simulations of HBB. They attended a pre-recorded lecture before being assigned to one of three instructors for the simulation lab. Participants completed a seven-point anonymous Likert-based questionnaire and a standardized Debriefing Assessment for Simulation in Healthcare Student Version (DASH-SV) Short Form. The primary outcome was the Objective Structured Clinical Exam (OSCE) grade. The trial is listed on ClinicalTrials.gov with the registration number NCT05257499.

Results: 47 participants completed the study, with similar baseline characteristics in each arm (gender, age, and class). The participants in both arms reported high levels of satisfaction and confidence, with no significant difference between the two arms. The DASH score over 7 was also similar in the online arm (6.27±0.26) compared to the in-person arm (6.55±0.13) (p=0.07). The mean OSCE score in the online arm (45.8±5.2) was comparable to the mean OSCE score in the in-person arm (41.3±5.0) (p=0.22). Both online and in-person participants failed the OSCE.

Conclusion: The survey responses conveyed that online simulation training is comparable to in-person simulation for the HBB course. Both online and in-person participants failed the OSCE most likely because they needed more training on HBB. This could be due to the fact that the material is too new to the students who needed more practice to pass the OSCE. Further research is needed to confirm these results and explore the long-term impact of online neonatal resuscitation training.

## Introduction

Birth asphyxia, a leading cause of neonatal death, contributed to the daily loss of around 6,500 newborns within one month of age in 2022, particularly in sub-Saharan Africa and southern and central Asia [1]. Simple interventions may prevent birth asphyxia: 5-10% of newborns require simple stimulation to help them breathe, 3-6% require bag-and-mask ventilation, and less than 1% require advanced resuscitation [2]. Launched by the American Academy of Pediatrics (AAP) in 2010, the Helping Babies Breathe (HBB) course teaches these primary interventions to healthcare professionals in low-resource settings [3]. Since then, the implementation of the HBB course has reduced the risk of neonatal mortality rate by up to 47% in lower and middle-income countries (LMIC) [4].

The HBB course serves as a model for other training programs. It includes innovative learning strategies, peer-to-peer learning, and visual educational material accompanied by low-cost realistic simulators [5]. Many studies have been exploring ways to improve the course by implementing different delivery modes, including virtual reality (VR) and the addition of videoconferencing [6,7].

The Covid-19 pandemic has disrupted HBB training and other medical education branches [8]. Virtual learning has become the norm and is expanding in medical training programs [9,10]. Several studies have supported its efficiency in teaching surgical procedures, techniques, and suturing [11-16]. However, the literature has been lacking regarding the virtual delivery of HBB.

Some studies have addressed the issue of tele-education in neonatal resuscitation [17,18]. However, only two pilot studies investigated the feasibility of virtual training in the HBB course [19,20]. The aim of this study is to evaluate the effectiveness of online debriefing compared to traditional face-to-face debriefing in the HBB course for nursing and medical students. Specifically, the study seeks to determine differences in OSCE scores between students who receive online versus in-person debriefing, compare their levels of satisfaction and confidence, and assess the feasibility and acceptability of online debriefing in HBB training. This study is one of the largest trials tasked to compare online and in-person training of HBB among medical and nursing students.

## Materials and methods

Design

The study design consists of a two-arm parallel randomized non-inferiority controlled trial. The control arm received an in-person debriefing, while the intervention arm received a virtual debriefing during HBB training. Participants were invited through emails and posters. The study was granted an expedited review by the Institutional Review Board (IRB). The trial has been listed on ClinicalTrials.gov with the registration number NCT05257499.

Participants

We included medical students and second-year nursing students. Participants with prior knowledge of neonatal resuscitation were excluded from this study by assessing their prior training in a neonatal resuscitation program (NRP) or HBB course or involvement with the Lebanese Red Cross. The trial was conducted at the American University of Beirut, Lebanon. Informed consent was obtained from all participants prior to enrollment, and they were informed that they could withdraw from the study at any time. All participants were informed that participation in this session was voluntary and would not affect their academic records.

Sample size

According to a previous HBB class given in the 2020 fall semester, OSCE grades varied between 80 to 100 over 100, with a standard deviation of 6. We calculated the sample size for non-inferiority to detect a 4% difference in OSCE scores between the two arms, assuming a power of 0.8 and α = 0.05. The initial sample size calculated was 56 (28 in each arm). After accounting for 20% loss to follow-up, the final sample size is 68 total participants.

Randomization

The participants were randomly assigned through a computer-allocated sequence to either the online or in-person simulation. FN, who was not involved in the recruitment, training or data collection process, generated the random sequence, and PK enrolled and assigned participants to the intervention groups while being concealed from the allocation process. All participants attended a 40-minute pre-recorded lecture about the basic theory of neonatal resuscitation, including a 10-minute online video depicting all the steps of the HBB course [21].

Then, they were divided into groups of 2-5 people and assigned one of three HBB instructors for the simulation lab according to the instructor’s availability. The certified instructor TG trained the other two instructors, PK and VM, on the proper administration of the HBB simulation. Prior to administering the simulation, PK and VM attended several training sessions and received specific instructions to ensure standardized delivery of the HBB simulation. The simulation course focused on the principles of stimulation and bag-mask ventilation using a NeoNatalie simulator (Laerdal Group, Stavanger, Norway).

**Figure 1:**
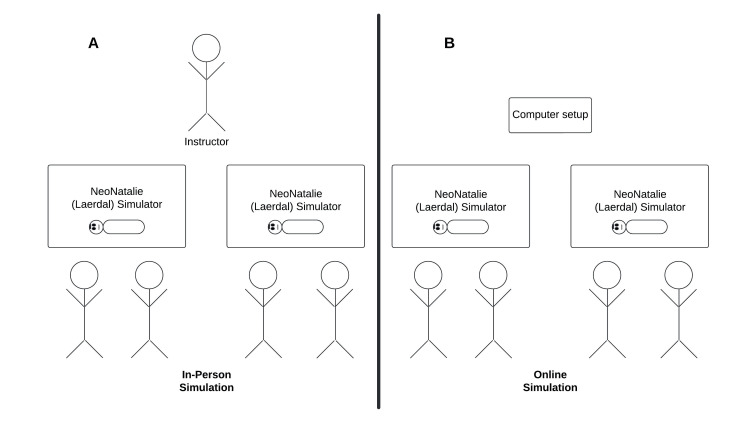
Comparison of in-person simulation setup (A) and online simulation setup (B) Depending on the assignment, the instructor presented the simulation either online or in-person. The simulation course focused on the principles of stimulation and bag-mask ventilation using a NeoNatalie simulator (Laerdal Group, Stavanger, Norway). In the control arm, the participants attended the simulation lab with the instructor in the same room (A). In the intervention arm, the instructor and the trainees were in two separate areas and interacted through Zoom (B). Figure credit: Peter Kfoury

Depending on the assignment, the instructor presented the simulation either online or in-person. In the control arm, the participants attended the simulation lab with the instructor in the same room. In the intervention arm, the instructor and the trainees were in two separate areas and interacted through Zoom™. Each participant attended one simulation lab, which took less than an hour (Figure 1). In the simulation sessions, every participant was tasked to perform a minimum of two simulation scenarios. The duration of the study, during which HBB training was conducted and tested, spanned two months, starting in mid-November 2022. The participants were provided with an online video about HBB simulation, posted by the Global Health Media Project as study material for the OSCE [21].

 

Outcomes

Following the hands-on simulations, on the same day, the participants completed a seven-point anonymous Likert-based questionnaire, comprising 17 items, to assess their level of satisfaction and confidence with the course (Appendix A), accompanied by the standardized Debriefing Assessment for Simulation in Healthcare Student Version (DASH-SV) Short Form [22]. During December and January 2022, both groups were assessed using the Objective Structured Clinical Exam (OSCE) within one to two months of learning. The OSCE was graded over 100 with a passing grade of 80%. The OSCE score was calculated as an average score between two blinded examiners (LC and FM) who assessed the participants’ performance through a de-identified recorded video. LC and FM were not involved as trainers in the study. The video was de-identified by concealing faces, identifiable bracelets, or watches; names were replaced by the study ID number. The primary outcome was the OSCE grade, while the level of satisfaction, confidence, and DASH score were secondary outcomes.

Statistical methods

Two research assistants entered the data into two separate datasheets. Following data entry and cleaning, the Likert scale questions were divided into three main scaled categories: Satisfaction, Confidence, and DASH. The Satisfaction category contained six questions, the confidence scale contained five, and the DASH is a 6-question survey. Each question is a seven-point Likert scale.

The primary outcome, secondary outcome, and sub-group analyses were assessed using a two-sample t-test since they were normally distributed, as shown through plotting and testing with the Kolmogorov-Smirnov test and the Shapiro-Wilk test. Chi-square test was used to assess the demographic differences between the two groups. Fisher’s exact test was used to compare the OSCE scores between the different instructors of the simulations. The mean differences were reported with 95% confidence intervals, and the statistical significance level was set at a p-value less than 0.05. All statistical analyses were performed in SPSS® version 24.

## Results

**Figure 2:**
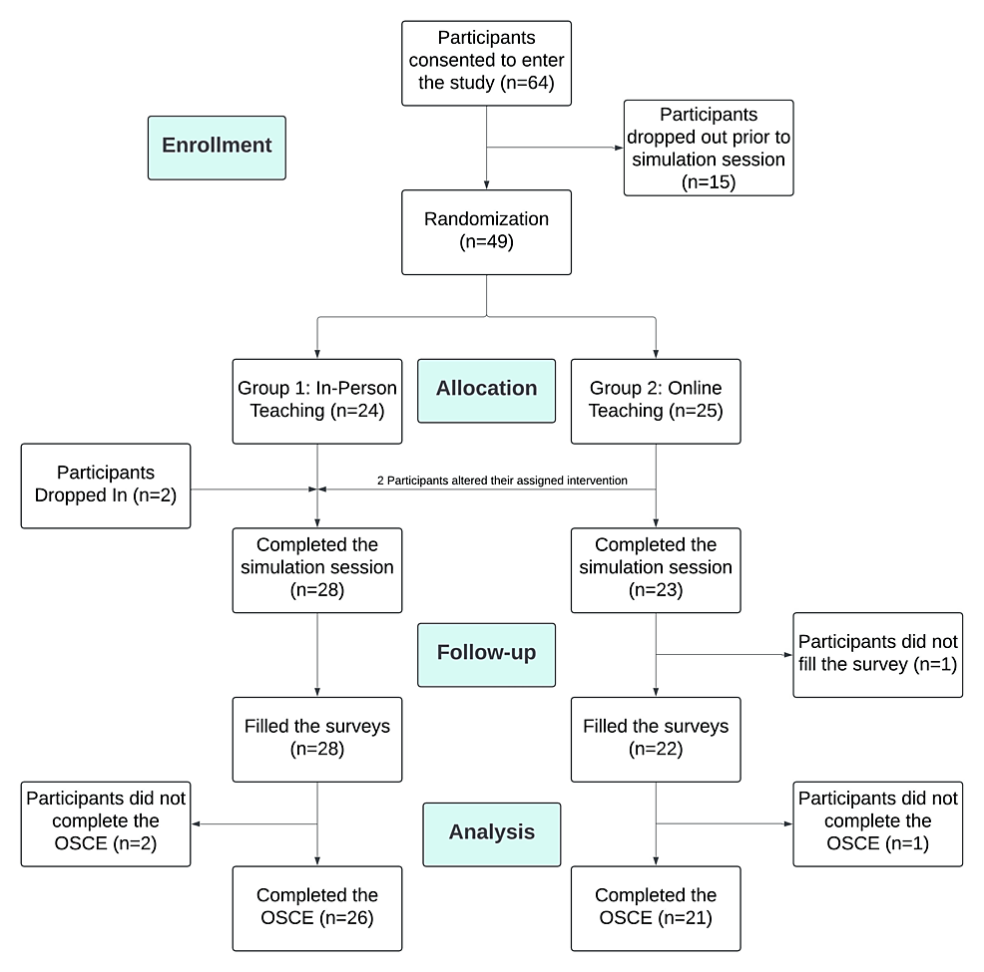
Trial CONSORT Flow Diagram This flowchart depicts the progress of participants in a study comparing in-person and online debriefing. Out of 64 consented participants, 49 were randomized: 24 to in-person debriefing and 25 to online debriefing. Two participants altered their assigned intervention, resulting in 28 completing in-person and 23 completing online simulation sessions. Surveys were completed by 28 in the in-person group and 22 in the online group. Ultimately, 26 in-person and 21 online participants completed the OSCE.

A total of 64 students agreed to participate in the study, 15 were lost to follow-up before their assignment (Figure 2). Thus, 49 participants were randomized into in-person training (n=24) or online training (n=25). However, two participants attended an in-person simulation session instead of their assigned online simulation. Two nursing students dropped in on the study and attended an in-person simulation session without randomized assignment.

Baseline characteristics of the population prior to the OSCE were similar in both groups (Chi-square test, p=0.98), including gender, age, class, and faculty (Table 1). The time elapsed between the simulation session and the OSCE was not statistically different between both groups, with the in-person teaching averaging 36.0±9.4 days and the online teaching averaging 24.3±10.1 (p=0.105). The participants were not equally distributed among instructors, with TG instructing 28 participants compared to PK and VM, who instructed 19 participants in total (Fisher's Exact Test, p<0.001). PK and VM instructed 17 participants in the face-to-face simulation while only teaching two participants online.

**Table 1:** Baseline characteristics of the population during the OSCE OSCE: Objective Structured Clinical Exam; MOE: Margin of error; MD: Medical degree; RN: Registered nurse

Actual Group		In-Person n=26 (55%)	Online n=21 (45%)	Total n=47 (100%)	p-value
Gender	Male	10 (38%)	8 (38%)	18 (38%)	0.98
	Female	16 (62%)	13 (62%)	29 (62%)	
Instructors	TG	9 (35%)	19 (90%)	28 (60%)	<0.001
	PK	10 (38%)	2 (10%)	12 (26%)	
	VM	7 (27%)	0	7 (15%)	
Class	MD student	16 (62%)	13 (62%)	29 (62%)	0.98
	Med 1	2	2	4	
	Med 2	5	3	8	
	Med 3	6	7	13	
	Med 4	3	1	4	
	RN student	10 (38%)	8 (38%)	18 (38%)	

OSCE scores (Table 2) in both groups were below the passing threshold: participants failed to meet the standards set out by our two blinded examiners.

**Table 2:** Participants’ OSCE score stratified by gender, instructor, and faculty N/A: Not available

		Mean OSCE Score (SD)		t-value	p-value
		In-person (n=26)	Online (n=21)		
Gender	Male	39.3 (16.8)	46.3 (14.9)	0.923	0.369
	Female	42.6 (10.0)	45.5 (10.0)	0.777	0.444
Instructor of Simulation	TG	45.0 (12.3)	45.7 (12.2)	0.141	0.885
	PK	41.4 (13.7)	46.3 (8.8)	0.476	0.645
	VM	36.5 (12.4)	N/A	N/A	N/A
Student Class	Nursing	33.7 (8.0)	38.9 (9.6)	1.255	0.219
	Medical	46.1 (13.1)	50.00 (11.2)	0.850	0.402
Total		41.3 (12.8)	45.8 (11.8)	1.240	0.221

**Figure 3:**
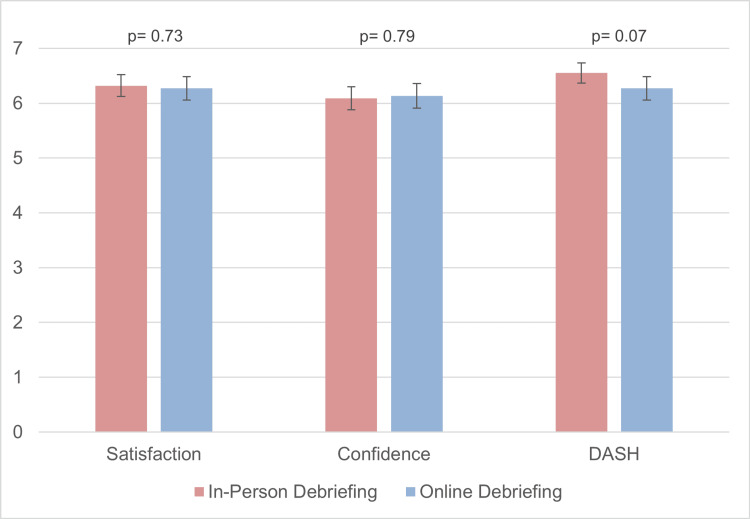
Comparison of mean Likert score in satisfaction, confidence, and DASH questionnaire between online and in-person learning The mean Likert scores for satisfaction, confidence, and DASH questionnaire responses are comparable between the online (n=22) and in-person (n=28) learning groups. Subcategories show no statistically significant differences between online and in-person teaching.

The mean Likert score for each question is similar for both online (n=22) and in-person (n=28) groups. Subcategorizing these questions into the satisfaction, confidence, and DASH scores shows no statistical difference between online and in-person (Figure 3). Participants receiving in-person learning have a mean satisfaction score of 6.32 ± 0.20 compared to a mean score of 6.27 ± 0.20 for the online group (p=0.73). Those instructed face-to-face received an average confidence score of 6.09 ± 0.20 compared to an average of 6.13 ± 0.22 for the online group (p=0.79). The DASH score is also comparable in both groups (p=0.07), with a mean score of 6.55 ± 0.13 for the in-person group and a mean score of 6.27 ± 0.26 for the online group.

## Discussion

This study compares the effectiveness of in-person to online HBB training for nursing and medical students. Unfortunately, both online and in-person participants did not meet the passing criteria set out by our examiners. This could be due to the fact that the material is too new to the students who needed more practice to pass the OSCE. Indeed, previous pilot studies have shown similar effectiveness of online and in-person HBB training for healthcare professionals in their OSCE performances [19,20].

Though the OSCE findings are disappointing, the surveys completed by the participants remain legitimate. The participants’ baseline characteristics were similar between the two groups, including gender, age, class, and faculty, which suggests that the randomization process was successful in creating comparable groups. The results display no significant difference between the online and in-person training groups’ participant satisfaction, confidence, and DASH scores; thus, there were no differences in the participants’ perceived ability to perform in clinical practice. Previous studies display ambiguous data on this topic; while some have shown a similar level of confidence and satisfaction [18,19], other studies have shown the opposite [16]. The decreased satisfaction and confidence may be attributed to connectivity problems and the distracting nature of learning a manual skill through a computer [16]. This problem could be solved by providing better connectivity and a better display of the procedure itself [23,24]. Therefore, our findings indicate that with optimal delivery, online HBB training may be an effective and satisfactory alternative to in-person training.

Several limitations must be considered when interpreting the results of this study. First, the loss to follow-up of 15 participants and the non-compliance of a few participants may have introduced some attritional bias and affected the validity of the results. Second, the uneven distribution of participants among instructors may have introduced variability in the teaching quality, potentially influencing the outcomes. Third, the study was conducted at a single institution, which may limit the generalizability of the findings to other settings or populations. Additionally, the reliance on self-reported measures of satisfaction and confidence may be subject to response bias. Finally, the OSCE scores, which were below the passing threshold for both groups, suggest that the training methods might not have been optimally effective, highlighting a potential mismatch between training delivery and evaluation criteria.

To ensure a consistent teaching protocol of HBB online training program investigations, organizers should standardize instructor training and balance participant distribution [25]. A robust follow-up system with reminders and incentives will minimize participant attrition. Investing in reliable technology and providing technical support will enhance virtual training effectiveness, while addressing connectivity issues through access to high-speed internet and necessary equipment [26,27]. Combining objective assessments with self-reported measures and aligning OSCE content with training objectives will provide a comprehensive evaluation of outcomes [28]. Establishing a continuous quality improvement framework will facilitate ongoing monitoring and iterative improvements, ensuring the training program remains effective and responsive to participant needs [29].

## Conclusions

This study indicates that online training of HBB is as effective as in-person training in terms of participant satisfaction and confidence, despite neither method resulting in a passing OSCE score for our cohort. These findings support the potential use of online training as an alternative to in-person sessions, especially when face-to-face training is not possible, e.g. due to a pandemic or travel costs. Further research with larger sample sizes, multi-center settings, and rigorous study designs are needed to confirm these findings and explore the long-term effects of online newborn resuscitation training.

## Appendices

Appendix 1

After carefully reading the information above, and having all your questions answered, and concerns addressed, do you voluntarily agree to participate in this study?

 Yes

 No - Thank you for your time

If you agree to participate in this study, we will give you a copy of this form if you wish to contact the principal investigator/IRB office at AUB at a later stage.

Part 1:

Question 1: Did you complete all the required classes for the "Helping Babies Breathe" course?

 Yes

 No

If not, what part of the course did you not complete?

 The online lecture

 The group training session

 The personalized simulation lab with debriefing

 The OSCE

Question 2: What is the intervention group you belong to?

 Group A: Traditional face-to-face debriefing during simulation lab

 Group B: Online debriefing during the simulation lab

Part 2: Demographic and Academic standing:

Question 3: What gender do you identify as?

 Female

 Male

 Other

 I prefer not to answer

Question 4: What is the class you belong to?

 First Year Nursing

 Second Year Nursing

 Med 1

 Med 2

 Med 3

 Med 4

 Other

 I prefer not to answer

Question 5: How old are you? ____________

Question 6: Why did you choose to participate in the study? (You can check multiple answers)

 Learn a new Skill

 Mandatory University Course

 Gain a recognized certificate

 Meet new people (nurses, medical students)

 Other ______________

Part 3: Level of satisfaction:

The following statements assess your level of satisfaction after the simulation and instructor’s debriefing. Please enter the number that best corresponds to the statements below:

1 - Extremely Disagree

2 - Disagree

3 - Slightly Disagree

4 - Neutral

5 - Slightly Agree

6 - Agree

7 - Extremely Agree

Question 7: The simulation was realistic

Question 8: I feel more comfortable assisting in the birth of a child

Question 9: I enjoyed the format of the personalized debriefing session

Question 10: The simulation session was effective

Question 11: The debriefing session helped me understand the appropriate actions to take when faced with a newborn

Question 12: The simulation session was enjoyable

Part 4: Level of Confidence:

The following statements assess your level of confidence after the simulation and instructor’s debriefing. Please enterthe number that best corresponds to the statements below:

1 - Extremely Disagree

2 - Disagree

3 - Slightly Disagree

4 - Neutral

5 - Slightly Agree

6 - Agree

7 - Extremely Agree

Question 13: After the simulation session, I feel more confident in my medical knowledge as it related to helping babies breathe

Question 14: After the simulation session, I feel more confident in managing a newborn suffering from asphyxia

Question 15: After the simulation session, I can plan ahead the necessary actions to take in neonatal resuscitation

Question 16: After the simulation session, I can demonstrate the step-by-step approach required to help babies breathe

Question 17: After the simulation session, I can use what I have learned to take care of neonates born outside a hospital

Part 5: Personal Suggestions concerning the course:

What improvements would you suggest for the overall presentation of the HBB course?
